# Editorial: New and current perspectives on social comparisons and their effects, volume II

**DOI:** 10.3389/fpsyg.2026.1825567

**Published:** 2026-05-07

**Authors:** Sviatlana Kamarova, Elif Nilay Ada, Masato Kawabata

**Affiliations:** 1 Sydney School of Health Sciences, The University of Sydney, Camperdown, NSW, Australia; 2 Sport Sciences Faculty, Mersin Universitesi, Mersin, Türkiye; 3 College of Sport and Wellness, Rikkyo University, Niiza, Saitama, Japan

**Keywords:** effects, mechanisms, motivation, outcomes, social comparison

Social comparison remains one of the most pervasive and consequential processes in human judgment and behavior (Caliskan et al., 2024; Festinger, 1954; Matthews and Kelemen, 2024). People are used to evaluate their abilities, opinions, and performance by referencing others, often automatically, yielding downstream effects that range from motivation and risk-taking to affective reactions and wellbeing (Lalot and Houston, 2025; Mussweiler et al., 2004; Sheffler and Cheung, 2024; Sun et al., 2023). Building on the success of the first Research Topic on this topic (Kamarova et al., 2021), the current second volume brings together empirically rigorous articles that promote the field forward on mechanisms, boundary conditions, and applied contexts for social comparison effects. The four contributions presented here span cognitive-perceptual accounts, motivational pathways, affective and growth-related outcomes, and real-world communication environments, offering a coherent yet diverse picture of current trajectory of social comparison science.

Using experimental design, Stinson et al. disentangled two often conflated explanations for egocentric biases in effort self-evaluation judgments: attributional accounts (self-serving explanations) and differences in information availability when acting vs. observing. By the direct manipulations of the stimuli, their findings showed that participants systematically evaluated tasks they performed as harder compared to the observed tasks, the effect that was strengthened with objective task difficulty increase. Therefore, the study demonstrated that sensory information asymmetry can drive egocentric tendencies in effort comparative judgement, outside of attributional bias.

Nie et al. examined the impact of downward social comparison on adversarial growth, defined as positive psychological change following hardship. Downward social comparisons predicted greater growth in adults with recent adversities through two mediators: self-acceptance and gratitude. The benefits were strongest for individuals high in interpersonal sensitivity. The study contributes to the literature through presenting evidence on adaptive meaning-making and self-recovery, building resilience rather than merely buffering more known effects of ego-threat (Stapel and Suls, 2004; Taylor and Lobel, 1989). In contrast, Zetian et al. examined the dual pathways of curiosity drive and downward social comparison in game advertising. Across five experiments the study extended knowledge on social learning (Bandura and Walters, 1977; Chatzisarantis et al., 2024; Schunk and DiBenedetto, 2020) into contemporary applied digital ecosystems. They identified a dual pathway mechanism operationalized where, on one side, information gaps trigger a curiosity drive that motivates exploration (play intent), while downward social comparisons boost perceived relative competence and competitive motivation, on the other. These evidence offers comparative tools to nudge one's attention and behavior.

Finally, Wang et al. investigated the link between social comparison orientation and domain-specific risk-taking through exploration of the mediating role of two dimensions of trait competitiveness. They found that upward performance sensitivity (an ability focused comparison mindset) does not uniformly escalate risk, but the competitiveness nature (determination to win at all costs as opposed growth oriented) effects one's risk-taking. The study contributes by shedding light on the competitiveness decision-making in the context of risk-taking.

Together, these contributions emphasize the role of social comparison effects that are driven by perceptual information asymmetries (Stinson et al.), motivational channels operationalized via competitive orientations (Wang et al.), and affective processes (gratitude and self-acceptance) (Nie et al.), that can be independently or synergistically activated in the real context (Zetian et al.). The volume thus answers long standing calls to move beyond surface level upward/downward distinctions toward process level explanations. Specifically, the Research Topic offers actionable strategies operationalized through task or message structure, domain to shape social comparison outcomes, where environments invite or constrain comparison processes. Importantly, this Research Topic suggests that comparisons are not inherently harmful or helpful. Downward comparison can be defensive in some settings, yet it may also offer recovery by gratitude and self-acceptance after adversity. The eventual effect of social comparison is essentially contingent on mediating dispositional mindsets and contextual cues (competitiveness and risk-taking).

This volume advances a more nuanced and context-sensitive science of social comparison. By triangulating perceptual inputs, motivational dispositions, affective regulators, and applied communication settings, the articles collectively demonstrate how comparison is entangled into cognition and culture. We hope this Research Topic stimulates further integrative work to: (i) clarify mechanisms with precision, (ii) test ethically mindful interventions, and (iii) leverage the adaptive potential of comparison while minimizing its risks.
